# The role of the bone microenvironment in regulating myeloma residual disease and treatment

**DOI:** 10.3389/fonc.2022.999939

**Published:** 2022-08-22

**Authors:** Terry G. Dadzie, Alanna C. Green

**Affiliations:** Weston Park Cancer Centre and Mellanby Centre for Musculoskeletal Research, Department of Oncology and Metabolism, The Medical School, University of Sheffield, Sheffield, United Kingdom

**Keywords:** myeloma, bone, dormancy, bone microenvironment, cancer dormancy, osteoblast (OB), axl

## Abstract

Multiple myeloma is an incurable haematological cancer. The increase in targeted therapies has improved the number of myeloma patients achieving a complete response and improved progression-free survival following therapy. However, a low level of disease or minimal residual disease (MRD) still persists which contributes to the inevitable relapse in myeloma patients. MRD has been attributed to the presence of dormant myeloma cells and their subsequent reactivation, which is controlled by the microenvironment and specialised niches within the bone marrow. This contributes to the evasion of the immune system and chemotherapy, eventually leading to relapse. The growth of myeloma tumours are heavily dependent on environmental stimuli from the bone marrow microenvironment, and this plays a key role in myeloma progression. The bone microenvironment also plays a critical role in myeloma bone disease and the development of skeletal-related events. This review focuses on the bone marrow microenvironment in relation to myeloma pathogenesis and cancer dormancy. Moreover, it reviews the current therapies targeting the bone microenvironment to treat myeloma and myeloma bone disease. Lastly, it identifies novel therapeutic targets for myeloma treatment and the associated bone disease.

## Introduction

Multiple myeloma is a malignant disease of plasma cells that undergo clonal proliferation in the bone marrow (BM) (1). Estimates from Global Cancer Incidence, Mortality and Prevalence (GLOBOCAN) indicated that myeloma accounted for 0.9% of patients diagnosed with cancer globally in 2018 (2). Complications derived from myeloma often constitutes hypercalcemia, renal failure, anaemia, and bone lesions, also referred to as the CRAB criteria (2). Myeloma treatment options have vastly increased and improved in recent years. This reflects a 40.3% increase in 5 years survival, from 6% in 1950-1954 to 46.3% in 2011-2017 (3). Despite this, myeloma remains incurable for the vast majority of patients (4). Since the implementation of the cytotoxic drug Melphalan in 1962, the landscape of myeloma treatments has evolved into combination therapies using different classes of drugs leading to improved survival (5). Treatments include proteasome inhibitors, immunomodulatory drugs, monoclonal antibody agents, among others. In patients deemed fit, a high dose of chemotherapy is administered followed by autologous stem cell transplant (6). The main goal for the clinical management of myeloma is to achieve operational cure. To be classified as ‘operationally cured’ patients need to maintain a stable complete response, with a low detectable level of disease. This plateau phase can last for months-years, and during this period patients may undergo maintenance therapy or no treatment (4). However, given most patients experience an inevitable risk of relapse within 10 years (4), the challenge now is to develop novel treatments that can eradicate residual disease. In myeloma, disease heterogeneity means no one therapy is effective against all myeloma cells, but the difficulty in eradicating the tumour also lies in the phenomenon of cancer dormancy. The BM microenvironment (BMME) plays a critical role in regulating myeloma dormancy and chemotherapy evasion. This review will discuss the roles of the BMME in regulating dormancy and the potential for therapeutically targeting the niche to prevent relapse and restore bone homeostasis in myeloma.

## Detection of minimal residual disease

The treatment of myeloma patients aims to balance aggressive multidrug induction treatment in order to induce a deep response whilst also minimising adverse effects and maintaining quality of life (7). The depth of response has been strongly correlated to progression-free survival (PFS) and overall survival (OS) (7). Traditionally, complete response (CR) has been used as a prognostic marker for OS and PFS following myeloma treatment (7). However, with increased usage of triple combination regimens, CR achievement is relatively common. Nonetheless, relapse remains a predicament, thus indicating the requirement of a deeper response criterion (8).

Through the incorporation of sensitive detection methods, MRD-negativity has been indicated to be a more favourable prognostic marker in terms of OS and PFS (9, 10). For a myeloma patient to be determined MRD-negative, bone marrow measurements should indicate no detectable myeloma cells within at least 100,000 normal cells (6, 11). The determination of MRD-negativity with sensitive methods including next-generation flow cytometry (NGF) and next generation sequencing (NGS) were shown to have a higher accuracy in predicting PFS compared to 4–8-colour flow cytometry due to higher sensitivity levels (12). Currently, flow cytometry is the most widely used despite being less sensitive than NGS and NGF (13, 14). This is mainly attributed to lower cost, shorter detection time and accessibility (15). The lower sensitivity of standard flow cytometry means the risk of not detecting residual myeloma cells is higher and explains why patients relapse even after having no detectable disease. A key limitation with MRD detection is that current techniques use samples obtained from a BM biopsy. This does not consider the high heterogeneity in disease distribution of myeloma throughout the skeleton and the existence of extramedullary disease. Therefore, conventional MRD detection is often paired with imaging studies using PET/CT or MRI, which can evaluate MRD beyond the BM (15). MRD-negativity in both imaging and NGF techniques has improved survival outcomes, when compared to patients with detectable disease in one or both of these measurements (16).

## The bone marrow microenvironment

The BMME of myeloma is categorised into distinct compartments which can broadly be defined as the perivascular niche and endosteal niche. The BMME is home to hematopoietic cells, osteoblast lineage cells, osteoclasts, adipocytes, fibroblasts, among other cell types and non-cellular components. Together, the niche supports the healthy production of blood cells and maintains bone homeostasis (17, 18). In myeloma, the BMME is implicated in mediating myeloma cell survival, proliferation, and drug resistance (19–25). Whilst the vascular niche is implicated in myeloma pathogenesis, this review will focus on the endosteal niche and its role in regulating myeloma dormancy.

## Myeloma pathogenesis and bone disease

**Figure 1:**
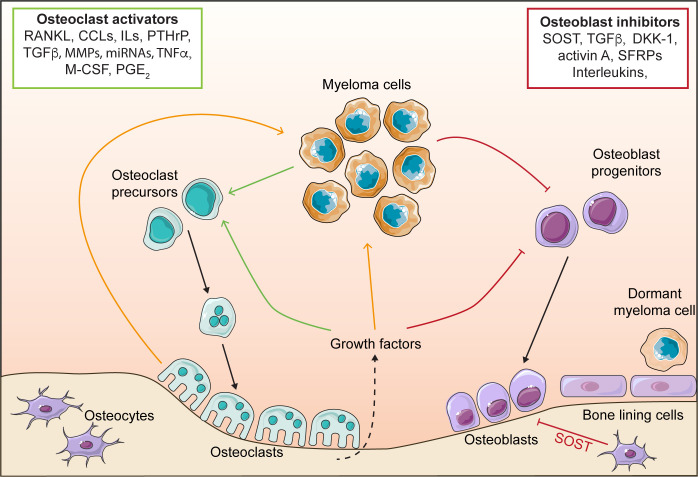
The vicious cycle in myeloma. Myeloma cells release osteoclast activating factors, which stimulate the formation of osteoclasts and increase bone resorption, leading to bone destruction. Myeloma cells also release osteoblast inhibitory factors that block osteoblast differentiation, and preventing bone formation. Osteoclasts release factors that stimulate myeloma growth. Elevated bone resorption leads to release of growth factors from the bone matrix (e.g. TGFβ) which further stimulate osteoclastogenesis and myeloma cell growth, whilst simultaneously inhibiting osteoblasts. Osteoblasts and bone lining cells promote myeloma dormancy. receptor activator of nuclear factor κ-B (RANKL), CC chemokine ligands (CCLs), interleukins (IL), parathyroid hormone-related protein (PTHrP), matrix metalloproteinases (MMPs), tumour necrosis factor α (TNFα), macrophage colony stimulating factor (M-CSF), prostaglandin E2 (PGE2), sclerostin (SOST), transforming growth factor β (TGFβ), Dikkopf protein 1 (DKK1) and secreted frizzled-related proteins (SFRPs).

The pathogenesis of myeloma is facilitated by the direct and indirect interactions between myeloma cells and microenvironment cells (26–29). Of particular relevance to the development of myeloma bone disease and control of myeloma dormancy are osteoclasts, osteoblasts and bone lining cells (19, 26, 29–31). Myeloma cells cause the dysregulation of osteoclasts and osteoblasts, which is a key driver of myeloma bone disease (**Figure 1**). The consequence of altering bone cells, is the development of a system that drives progressive myeloma growth and bone destruction. This is termed the ‘vicious cycle’, where myeloma cells release factors that promote the formation of osteoclasts, this elevates bone resorption, releasing growth factors from the bone matrix that promote tumour growth and further dysregulation of bone cells. Simultaneously, myeloma cells inhibit osteoblast formation, thereby preventing formation of new bone and exacerbating the impact of the osteolytic bone disease. The vicious cycle and the many factors that stimulate osteoclasts and inhibit osteoblasts have been described previously in a number of reviews (26–29).

## Myeloma cell dormancy and the BMME

**Figure 2:**
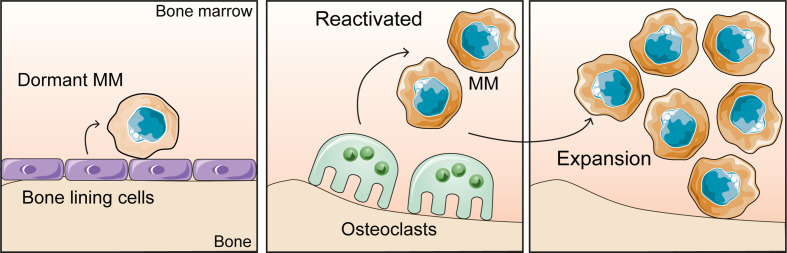
The bone microenvironment controls the myeloma growth dynamics. Dormant myeloma cells associate with bone lining cells and osteoblasts on the bone surface. Bone resorption by osteoclasts releases myeloma cells from dormancy and promotes tumour expansion.

The implications of the BMME on dormancy is an area of increasing interest. Dormancy occurs when myeloma cells enter a quiescent state (G_0_), where they are under reversible growth arrest (32). Myeloma cells become dormant and subsequently re-enter the cell cycle in response to extrinsic stimuli from the microenvironment (**Figure 2**) or in response to some therapies including bortezomib (33). The phenomenon of dormancy is problematic when it comes to eliminating myeloma, because whilst the cancer cells are quiescent, they are resistant to chemotherapy and radiotherapy. As a result these cells can persist in the bone marrow, and pose a challenge when it comes to eradicating residual disease (32). Therefore, the remaining dormant myeloma cells pose a threat of relapse when they are released from dormancy and re-enter the cell cycle. The localisation of dormant myeloma cells in the endosteal niche is reminiscent of the occupation of unique niches in the BM by hematopoietic stem cells (HSC), where it is proposed that myeloma cells take advantage of the same niche factors that support HSC quiescence (19, 20, 34, 35).

Lawson *et al.* demonstrated that in murine models, dormancy was induced in myeloma cells through the interaction of myeloma cells with osteoblasts or bone lining cells resident in the endosteal niche (19). In addition, there were indications that dormancy was maintained through the downregulation of genes which are essential for cell cycle progression and cell replication (19). Inversely, results obtained from mice that were induced with RANKL to increase osteoclastogenesis, showed that as the number of osteoclasts and subsequently bone resorption increased, the number of dormant myeloma cells decreased. Therefore, it can be inferred that the remodelling of the endosteal niche by osteoclasts causes the displacement of dormant myeloma cells from the niche and reactivates previously dormant myeloma cells (19).

Chen *et al.* supported the concept of quiescent myeloma cell occupancy in the osteoblast niche of the BM. *In vivo* studies conducted in irradiated NOD/SCID mice, which were simultaneously injected with CD34^+^ HSC and myeloma cells indicated that the occupation of HSC in the BM niche decreased as the number of myeloma cells injected increased (35). In addition, myeloma cell homing to the BM indicated that the osteoblastic niche was the primary site for PKH^+^ labelled myeloma cells (identifying non-dividing cells) occupation compared to the vascular niche (35). Further investigations using bone biopsies of myeloma patients indicated that quiescent PKH^+^ myeloma cells expressed a higher number of a gene named TRIM44 compared to proliferating myeloma cells. The upregulation of TRIM44 was determined to maintain myeloma cells to a quiescent state and reduce cell proliferation (35). Through its deubiquitination function, TRIM44 stabilised and prevented the proteasomal degradation of hypoxia-inducible factor 1α (HIF-1α) under hypoxic and normoxic conditions (35). This revealed that HIF-1α expression and response to hypoxia regulate myeloma dormancy, similarly the role in HSC quiescence in the BM as well as tumour dormancy (36–38).

Lastly, Lawson *et al.* identified the upregulation of AXL (TAM receptor tyrosine kinase member) in dormant myeloma cells. Khoo *et al.* then determined that the expression of AXL was upregulated in myeloma dormant cells (CD138^+eGFP^+1,1’-dioctadecyl-3,3,3′,3′-tetramethylindodicarbocyanine^hi^ (DiD^hi^)) *in vivo*. Furthermore, expression of AXL and Gas6, a ligand that interacts with AXL were induced when cocultured with osteoblasts (MC3T3 cells) in the BM and led to the retention of myeloma cells in dormant state (20). Administration of the AXL inhibitors Cabozantinib or BMS-777607 in mice with 5TGM1 tumours led to the reduction in dormant myeloma cells and an increase in reactivated cells. Which further substantiates AXL’s role in regulating the maintenance of dormant myeloma cells from the endosteal niche (20). Interestingly, the number of plasma cells containing AXL decreased as the disease progressed from MGUS to relapsed disease. This supports the notion that AXL maintains myeloma cells in a dormant state, and inhibition of AXL signalling causes myeloma cells to reactivate. Moreover, this identifies AXL as a potential biomarker for disease progression and distinguishes MGUS from myeloma patients (20). These results were substantiated by Waizenegger *et al.* as AXL was indicated to have higher expression levels in MGUS compared to myeloma patients (39). In addition, AXL expression in human cell lines (U266 and RPMI-8226) when cultured without osteoblasts were low, which was in line with Khoo *et al.* results showing osteoblasts support dormancy.

## Targeting the microenvironment in myeloma patients

Curative treatment in myeloma is hindered by the interactions between the BMME and myeloma cells, which facilitates dormancy, drug resistance and relapse. Therefore, alongside therapeutics that target myeloma cells it is also important to incorporate treatment strategies that can target the microenvironment to improve bone and tumour outcomes.

Anti-myeloma treatments will alleviate, to some extent, the bone disease indirectly by reducing tumour and hence lowering pro-osteoclastic and anti-osteoblastic factors. However, it is now recognised that some myeloma treatments also have direct effects on bone cells and have positive effects on myeloma bone disease. For instance, the front-line treatment bortezomib reduces bone resorption and increases bone formation in patients (40). Whilst the anti-tumour effects of bortezomib would certainly contribute to this, bortezomib also has direct bone anabolic and anti-osteoclastic activity. In human osteoclast precursors, bortezomib or the bone-targeted bisphosphonate-tagged (BP-)bortezomib blocks RANKL-induced osteoclastogenesis, preventing osteoclast formation and resorption (41, 42). Treatment of neonatal mouse calvarial organ cultures with bortezomib elevated bone formation in a dose-dependent manner (43). This phenomenon was attributed to increased BMP-2 production in osteoblasts paired with decreased expression of DKK1 (43). Further investigated by Qian *et al.* found bortezomib stabilised β-catenin, thereby promoting osteoblast formation through Wnt-independent activation of pro-osteoblastic β-catenin signalling in primary mesenchymal stem cells (44). These findings are supported by *in vivo* studies showing stimulation of bone formation, increased bone volume and enhanced fracture repair in response to bortezomib or BP-bortezomib (42). Furthermore, treatment of mice bearing U266 myeloma tumours with bortezomib and lenalidomide healed existing bone lesions (45). The positive effects on bone indicate the of use of bortezomib may be particularly beneficial to patients with severe bone disease, in addition to targeting tumour cells. Considering the significant adverse effects caused by bortezomib, there may be a place for the use of BP-bortezomib in myeloma therapy, particularly in situations where the cancer is controlled but bone lesions are present, which warrants further study.

Given the role of osteoclasts in myeloma induced osteolysis, osteoclasts are a cellular target in the treatment of myeloma. Bisphosphonates were developed to delay bone disease progression (46). The bisphosphonates induce osteoclast apoptosis and are separated into non-nitrogen containing (e.g. clodronate) and nitrogen containing (e.g. zoledronate and pamidronate) (46). Although there are no direct, in human clinical trials comparing the three bisphosphonates, a meta-analysis indicated zoledronate, pamidronate and chlodronate to be non-inferior to each other in terms of OS and PFS (47). An MRC Myeloma IX study in 1960 newly diagnosed myeloma patients aged 18 and above determined zoledronate (27% of 981 patients) to be the superior drug in decreasing the incidence of skeletal-related events compared to clodronate (35% of 979 patients) during the 60-month study period (48). It is important to note that these findings may not be representative of all races as 97% of myeloma patients included in the study were White. Another anti-resorptive approved to treat myeloma is Denosumab, a monoclonal antibody to RANKL (49). In myeloma patients, Denosumab was noninferior to zoledronate in preventing skeletal related events and lower renal toxicity (49, 50). Thus, indicating denosumab’s usefulness in renal impaired patients. However, more recently the concern over the rebound bone loss and increased fractures following discontinuation of Denosumab in osteoporosis, which is an important consideration for use in myeloma (51, 52).

Whilst anti-resorptive agents are widely used to treat myeloma, there are no approved bone anabolic agents, even though osteoblast dysfunction in myeloma is well recognised. Some promising targets in development for bone anabolic therapy in myeloma include Dikkopf protein 1 (DDK1), sclerostin, activin A and transforming growth factor β (TGFβ). Romosozumab (anti-Sclerostin) was recently approved for the treatment of severe osteoporosis. In pre-clinical mouse models anti-sclerostin antibodies prevented bone loss and increased fracture resistance, which was further enhanced by combining with zoledronate (53, 54), making Romosozumab an attractive treatment for myeloma bone disease. Another promising target is TGFβR1 signalling, where an anti-TGFβ antibody (1D11) or a small molecule inhibitor to TGFβR1 (SD-208) prevent development of bone disease (45, 55, 56) and can repair existing bone lesions (45, 56). Treatment of myeloma patient bone marrow stromal cells with SD-208 also stimulated osteoblastic differentiation (45). Similarly the anti-DKK1 antibody BHQ880 protected against development of myeloma bone disease *in vivo* (57, 58) and inhibited tumour growth (59). DKK1 inhibitors are in phase 1/2 clinical trials inlcuding DKN-01 (NCT01711671) and BHQ-880 (NCT01337752). Results from a phase Ib trial on BHQ-880 showed a trend to increased bone mineral density, and whilst the phase II trial is complete no detailed results have been published. Furthermore, an inhibitor of Activin A (Sotatercept) was found to increase bone mineral density in myeloma patients in a phase IIa trial (NCT00747123) (60).

## Targeting dormant myeloma cells

There is currently a void in clinical strategies targeting dormant myeloma cells, which is likely key to developing a curative treatment for myeloma. Studies by Lawson *et al.*, Khoo *et al.* and Chen *et al.*, have pioneered the field in understanding microenvironmental control of myeloma dormancy (19, 20, 34, 35). Through these studies a number of characteristics that are unique to dormant myeloma cells have been identified, including elevated expression of Axl, Fcer1g, Csf1r, Sirpa (19, 20) and TRIM44 (35). Notably, this provides insight into possible molecular mechanisms behind dormancy that can be exploited to develop a new class of myeloma treatment. Indeed this has been explored experimentally, where inhibiting AXL with cabozantinib or BMS777607 releases myeloma cells from dormancy (20), potentially sensitising cells to chemotherapeutic agents that target dividing cells. However, clinically, whether this is a safe and effective approach is uncertain as tumour growth reactivation would be disadvantageous if chemotherapeutics were subsequently ineffective, which is a realistic concern considering the heterogeneity of myeloma. Interestingly, combining bortezomib with the AXL inhibitor cabozantinib prolonged survival in 5TGM1-bearing mice (61). Whether this means that cells are in some way more vulnerable to treatment intervention following AXL inhibitor-driven release from dormancy requires further study.

In conclusion, the BMME significantly impacts both treatment outcomes, myeloma cell dormancy and bone disease. Incorporating bone anabolic agents to treat myeloma bone disease is important for facilitating healing of existing bone lesions and would improve the quality of life for myeloma patients. Furthermore, the risk of relapse in myeloma highlights the need further for new myeloma treatments that kill and eradicate dormant myeloma cells safely and effectively. Therefore, targeting the interplay between the myeloma cells and the BMME is vital for improving myeloma therapy. This will require more detailed characterisation of the dormancy niche to identify the specific osteoblast lineage cells that support dormancy, given the heterogeneity of bone lining cells in the microenvironment (62). Identification of the mechanisms that control dormancy induction, maintenance and release will reveal vulnerabilities of dormant myeloma cells that will assist in developing new targeted therapies for myeloma.

## Author contributions

The manuscript was written by TD and AG. All authors read and approved the final manuscript.

## Funding

This paper was published with support from the University of Sheffield Institutional Open Access Fund. AG is supported by a Sheffield Hospitals Charity Project Grant number: 202101.

## Conflict of interest

The authors declare that the research was conducted in the absence of any commercial or financial relationships that could be construed as a potential conflict of interest.

## Publisher’s note

All claims expressed in this article are solely those of the authors and do not necessarily represent those of their affiliated organizations, or those of the publisher, the editors and the reviewers. Any product that may be evaluated in this article, or claim that may be made by its manufacturer, is not guaranteed or endorsed by the publisher.
